# Supplementary figures and images for: Temporal social network modeling of mobile connectivity data with graph neural networks
Source: PLoS One. 2025 Dec 10;20(12):e0335267. doi: 10.1371/journal.pone.0335267 (PMC12694809; doi:10.1371/journal.pone.0335267)

DySAT GCRN VGRNN rEdgeBank ROLAND

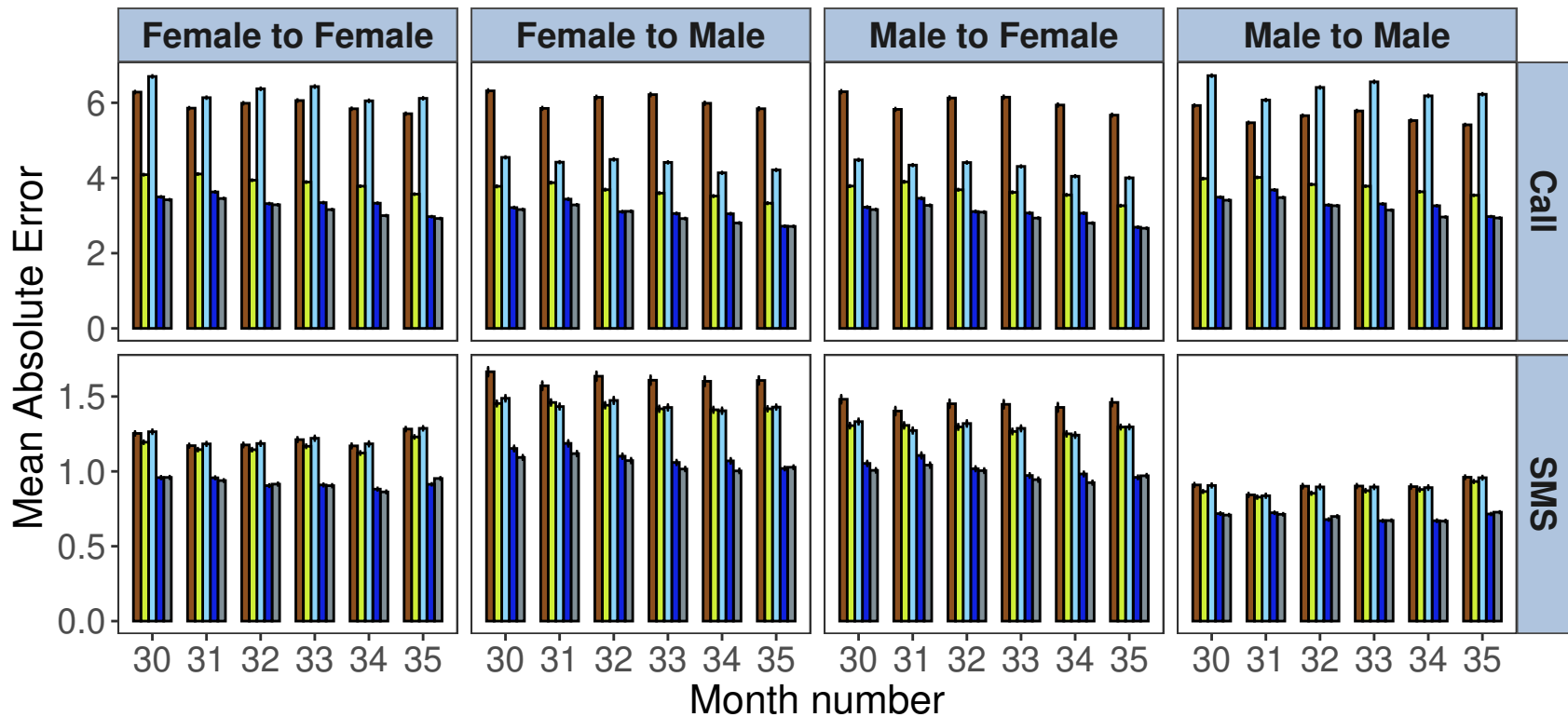

Supplement: S1 Fig — The MAEs calculated on the test data (last 6 months of the dataset) is shown with respect to gender of the source and the destination nodes. (PDF) [file pone.0335267.s001.pdf]

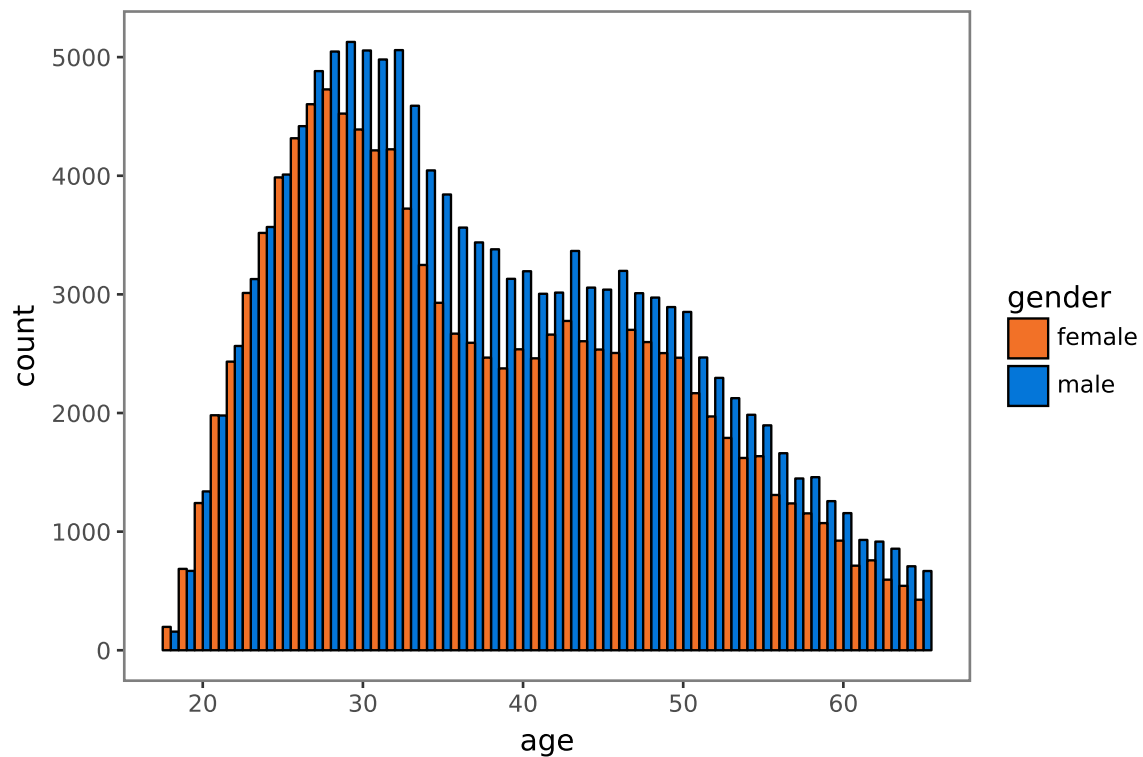

Supplement: S2 Fig — The distribution of the age of users for both males and females is shown. Users below the age of 18 and above 65 have not been considered for the present study. (PDF) [file pone.0335267.s002.pdf]
